# Correlation between the migration scale index and the number of new confirmed coronavirus disease 2019 cases in China

**DOI:** 10.1017/S0950268820001119

**Published:** 2020-05-19

**Authors:** Huijie Chen, Ye Chen, Zhiyong Lian, Lihai Wen, Baijun Sun, Ping Wang, Xinghai Li, Qiong Liu, Xiaoyun Yu, Ying Lu, Ying Qi, Shuo Zhao, Linlin Zhang, Xiaodan Yi, Fengyu Liu, Guowei Pan

**Affiliations:** 1 Department of Infectious Disease, Shenyang Center for Disease Control and Prevention, Shenyang 110031, Liaoning Province, China; 2 Department of Infectious Disease, Shenyang Sixth People's Hospital, Shenyang 110006, Liaoning Province, China; 3 National Health Laboratory, China Medical University, Shenyang 110122, Liaoning Province, China

**Keywords:** Coronavirus disease 2019, correlation, migration scale index

## Abstract

In late December 2019, patients of atypical pneumonia due to an unidentified microbial agent were reported in Wuhan, Hubei Province, China. Subsequently, a novel coronavirus was identified as the causative pathogen which was named SARS-CoV-2. As of 12 February 2020, more than 44 000 cases of SARS-CoV-2 infection have been confirmed in China and continue to expand. Provinces, municipalities and autonomous regions of China have launched first-level response to major public health emergencies one after another from 23 January 2020, which means restricting movement of people among provinces, municipalities and autonomous regions. The aim of this study was to explore the correlation between the migration scale index and the number of confirmed coronavirus disease 2019 (COVID-19) cases and to depict the effect of restricting population movement. In this study, Excel 2010 was used to demonstrate the temporal distribution at the day level and SPSS 23.0 was used to analyse the correlation between the migration scale index and the number of confirmed COVID-19 cases. We found that since 23 January 2020, Wuhan migration scale index has dropped significantly and since 26 January 2020, Hubei province migration scale index has dropped significantly. New confirmed COVID-19 cases per day in China except for Wuhan gradually increased since 24 January 2020, and showed a downward trend from 6 February 2020. New confirmed COVID-19 cases per day in China except for Hubei province gradually increased since 24 January 2020, and maintained at a high level from 24 January 2020 to 4 February 2020, then showed a downward trend. Wuhan migration scale index from 9 January to 22 January, 10 January to 23 January and 11 January to 24 January was correlated with the number of new confirmed COVID-19 cases per day in China except for Wuhan from 22 January to 4 February. Hubei province migration scale index from 10 January to 23 January and 11 January to 24 January was correlated with the number of new confirmed COVID-19 cases per day in China except for Hubei province from 22 January to 4 February. Our findings suggested that people who left Wuhan from 9 January to 22 January, and those who left Hubei province from 10 January to 24 January, led to the outbreak in the rest of China. The ‘Wuhan lockdown’ and the launching of the first-level response to this major public health emergency may have had a good effect on controlling the COVID-19 epidemic. Although new COVID-19 cases continued to be confirmed in China outside Wuhan and Hubei provinces, in our opinion, these are second-generation cases.

## Background

On 31 December 2019, Wuhan, China reported an outbreak of atypical pneumonia caused by SARS-CoV-2 and cases have been exported to other Chinese provinces, as well as internationally [1]. The atypical pneumonia caused by SARS-CoV-2 was tentatively named novel coronavirus pneumonia [2] by the National Health Commission of the People's Republic of China and the World Health Organization has named the disease coronavirus disease 2019 (abbreviated as ‘COVID-19’) on 11 February 2020 [3].

The virus has spread to all prefectures of Hubei province outside Wuhan and all provinces of China have also reported cases outside of Hubei province. Currently, most cases are related to Wuhan and others were caused by patients from Wuhan [4].

Following the confirmed COVID-19 cases reported in Zhejiang province, Guangdong province, Shanghai and other provinces in China reported COVID-19 cases one after another from 20 January 2020.

To control the epidemic of COVID-19, Wuhan Municipal People's Government issued a notice saying that citizens should not leave Wuhan for no special reason and the airport and train station from Wuhan corridor were temporarily closed from 23 January 2020 which called ‘Wuhan lockdown’. In addition, Wuhan's urban bus, subway, ferry and long-distance passenger transportation were suspended [5]. Zhejiang province launched first-level response to major public health emergencies from 23 January 2020 and subsequently other provinces, municipalities and autonomous regions in China launched first-level response to major public health emergencies one after another, which means movement of people among provinces, municipalities and autonomous regions was restricted [6].

The behaviour of the travellers leaving the city of residence for a short time and moving into other cities is migration [7]. Big data of migration comes from the massive positioning service data of the open platform and it recorded more types of spatial displacement, including airplane, high-speed rail, ship, coach and private car, so it theoretically has higher accuracy [8]. The migration scale index is not an absolute value of the number of travellers but is converted based on the absolute value of the number of travellers. We estimate one migration scale index is equal to about 56 137 travellers [8]. Migration scale index reflects the scale of the population migration from a city or a province, which can be compared horizontally among cities or provinces [9].

We conducted this study to explore the correlation between migration scale index and the number of confirmed COVID-19 cases. At the same time, we depict the effect of ‘Wuhan lockdown’ and Hubei province launching first-level response to major public health emergencies on the control of COVID-19 epidemic.

## Methods

### Data sources

Data of confirmed COVID-19 cases were extracted from the official websites of the National Health Commission of the People's Republic of China and Health Committee of provinces. Data of migration scale index were extracted from Baidu migration (http://qianxi.baidu.com/). The migration scale indexes of Wuhan and Hubei provinces from 1 January 2020 to 12 February 2020 were used in this study.

### Case definition

The diagnosis of COVID-19 is based on the diagnosis and treatment plan of pneumonia caused by novel coronavirus (trial version 5) established by the National Health Commission of the People's Republic of China [10].

### Pearson correlation analysis

SPSS 23.0 software was used for Pearson correlation analysis and the significance level used was *P* < 0.05. Based on the incubation period of illness from MERS and SARS coronaviruses, CDC believes that symptoms of COVID-19 infection occur within 2–14 days following infection [11]. The diagnosis and treatment plan of pneumonia caused by novel coronavirus (trial version 5) reports the longest incubation period of COVID-19 was 14 days [10] and due to the lag effect COVID-19 onset, we used 14 days' migration scale data from 1 January 2020 to 2 February 2020 and new confirmed cases per day to analyse the correlation. Considering that COVID-19 originated from Wuhan which is the capital city of Hubei province, then spread to Hubei province, an outbreak occurred in Hubei province [4] and following the confirmed COVID-19 cases reported in Zhejiang province, Guangdong province, Shanghai and other Chinese provinces reported COVID-19 cases one after another from 20 January 2020. Therefore, we performed two Pearson correlation analyses. They were Pearson correlation analysis between Wuhan migration scale index and the number of new confirmed COVID-19 cases in China except for Wuhan from 20 January to 2 February and Pearson correlation analysis between Hubei province migration scale index and the number of new confirmed COVID-19 cases in China except for Hubei province from 20 January to 2 February.

## Results

### Temporal pattern

**Fig. 1.**
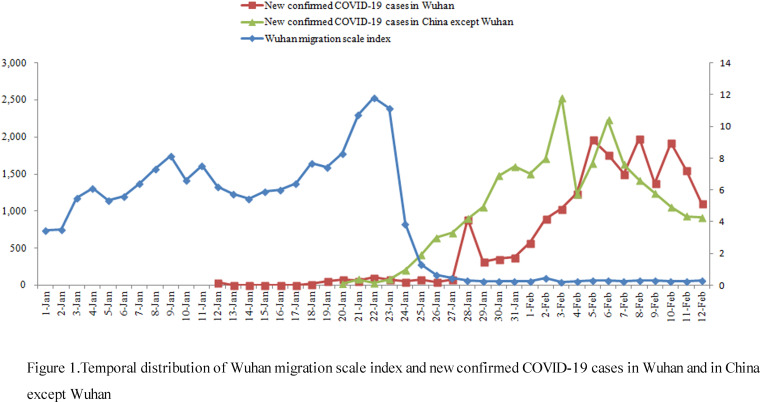
Temporal distribution of Wuhan migration scale index and new confirmed COVID-19 cases in Wuhan and in China except for Wuhan.

**Fig. 2.**
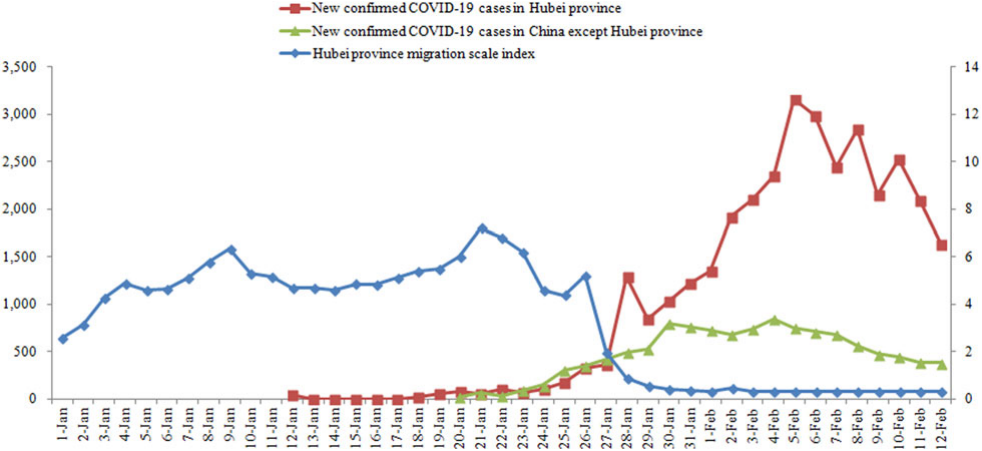
Temporal distribution of Hubei province migration scale index and new confirmed COVID-19 cases in Hubei province and in China except for Hubei province.

Since 23 January 2020, Wuhan migration scale index has dropped significantly (from 1st January to 12th February; *P* < 0.001) and since 26 January 2020, Hubei province migration scale index has dropped significantly (from 1st January to 12th February; *P* < 0.001). New confirmed COVID-19 cases per day in China except for Wuhan gradually increased since 24 January 2020, and showed a downward trend from 6 February 2020. New confirmed COVID-19 cases per day in Wuhan continued to increase since 27 January 2020, and maintained at a high level without a downward trend. New confirmed COVID-19 cases per day in China except for Hubei province gradually increased since 24 January 2020, and maintained at a high level from 24 January 2020 to 4 February 2020, then showed a downward trend. New confirmed COVID-19 cases per day in Hubei province continued to increase since 24 January 2020, and maintained at a high level without a downward trend (Tables 1 and 2, Figs 1 and 2).

**Table 1.** Wuhan migration scale index and new confirmed COVID-19 cases in Wuhan and in China except for Wuhan

	Wuhan migration scale index	New confirmed COVID-19 cases in Wuhan	New confirmed COVID-19 cases in China except for Wuhan
1 January	3.46		
2 January	3.52		
3 January	5.52		
4 January	6.10		
5 January	5.32		
6 January	5.60		
7 January	6.41		
8 January	7.34		
9 January	8.14		
10 January	6.62		
11 January	7.56		
12 January	6.22	41	
13 January	5.76	0	
14 January	5.46	0	
15 January	5.91	0	
16 January	6.00	0	
17 January	6.44	4	
18 January	7.71	17	
19 January	7.41	59	
20 January	8.31	77	21
21 January	10.74	60	77
22 January	11.84	105	33
23 January	11.14	81	80
24 January	3.89	51	202
25 January	1.30	77	405
26 January	0.66	46	640
27 January	0.43	80	705
28 January	0.32	892	897
29 January	0.26	315	1053
30 January	0.24	356	1477
31 January	0.24	378	1603
1 February	0.24	576	1503
2 February	0.46	894	1705
3 February	0.21	1033	2533
4 February	0.23	1242	1225
5 February	0.28	1967	1645
6 February	0.28	1766	2229
7 February	0.27	1501	1631
8 February	0.28	1985	1418
9 February	0.29	1379	1244
10 February	0.27	1920	1054
11 February	0.27	1552	931
12 February	0.29	1104	915

**Table 2.** Hubei province migration scale index and new confirmed COVID-19 cases in Hubei province and in China except for Hubei province

	Hubei province migration scale index	New confirmed COVID-19 cases in Hubei province	New confirmed COVID-19 cases in China except for Hubei province
1 January	2.56		
2 January	3.13		
3 January	4.26		
4 January	4.88		
5 January	4.58		
6 January	4.67		
7 January	5.11		
8 January	5.77		
9 January	6.32		
10 January	5.30		
11 January	5.16		
12 January	4.69	41	
13 January	4.69	0	
14 January	4.60	0	
15 January	4.86	0	
16 January	4.85	0	
17 January	5.10	4	
18 January	5.42	17	
19 January	5.49	59	
20 January	6.03	77	21
21 January	7.21	60	65
22 January	6.80	105	33
23 January	6.21	69	92
24 January	4.59	105	148
25 January	4.39	180	302
26 January	5.20	329	357
27 January	1.98	365	420
28 January	0.87	1291	498
29 January	0.54	840	528
30 January	0.40	1032	801
31 January	0.37	1220	761
1 February	0.33	1347	732
2 February	0.48	1921	678
3 February	0.34	2103	743
4 February	0.32	2345	842
5 February	0.31	3156	752
6 February	0.31	2987	712
7 February	0.30	2447	685
8 February	0.31	2841	562
9 February	0.32	2147	476
10 February	0.33	2531	443
11 February	0.32	2097	386
12 February	0.34	1638	381

### Pearson correlation analysis

Wuhan migration scale index from 9 January to 22 January, 10 January to 23 January and 11 January to 24 January was correlated with the number of new confirmed COVID-19 cases per day in China except for Wuhan from 20 January to 2 February. Hubei province migration scale index from 10 January to 23 January and 11 January to 24 January was correlated with the number of new confirmed COVID-19 cases per day in China except for Hubei province from 20 January to 2 February (Table 3).

**Table 3.** Pearson correlation analysis between migration scale index and the number of new confirmed COVID-19 cases per day from 20 January to 2 February in China except for Wuhan and in China except for Hubei province

Date range	*r* Value^a^	*P*^a^	*r* Value^b^	*P*^b^
1 January–14 January	0.484	0.080	0.526	0.053
2 January–15 January	0.256	0.377	0.301	0.296
3 January–16 January	−0.041	0.889	0.019	0.947
4 January–17 January	−0.186	0.524	−0.195	0.505
5 January–18 January	−0.034	0.909	−0.222	0.446
6 January–19 January	−0.043	0.884	−0.257	0.375
7 January–20 January	0.142	0.628	−0.121	0.681
8 January–21 January	0.443	0.113	0.217	0.457
9 January–22 January	0.642	0.013*	0.489	0.076
10 January–23 January	0.839	0.000*	0.821	0.000*
11 January–24 January	0.545	0.044*	0.763	0.002*
12 January–25 January	0.190	0.515	0.522	0.056
13 January–26 January	−0.170	0.561	0.304	0.291
14 January–27 January	−0.463	0.095	−0.156	0.595
15 January–28 January	−0.639	0.014	−0.412	0.143
16 January–29 January	−0.745	0.002	−0.599	0.024
17 January–30 January	−0.812	0.000	−0.809	0.000
18 January–31 January	−0.858	0.000	−0.899	0.000
19 January–1 February	−0.846	0.000	−0.944	0.000
20 January–2 February	−0.785	0.001	−0.942	0.000

a Refers to Pearson correlation analysis between Wuhan migration scale index and the number of new confirmed COVID-19 cases in China except for Wuhan per day from 20 January to 2 February.

b Refers to Pearson correlation analysis between Hubei province migration scale index and the number of new confirmed COVID-19 cases in China except for Hubei province per day from 20 January to 2 February.

‘*’ means significant at the level of *P* < 0.05.

## Discussion

In December 2019, a cluster of acute respiratory illness, now known as novel coronavirus disease (COVID-19), occurred in Wuhan, Hubei province, China [12–16]. The disease has rapidly spread from Wuhan to other areas. As of 12 February 2020, a total of 44 763 COVID-19 cases in China have been confirmed. Internationally, cases have been reported in 24 countries and five continents [17]. Although the origin of the SARS-CoV-2 is still being investigated, current evidence suggests spread to humans occurred via transmission from wild animals illegally sold in the Huanan Seafood Wholesale Market [18, 19].

The SARS-CoV-2 has impacted multiple countries, caused severe illness and sustained person-to-person transmission making it a concerning and serious public health threat [11]. Previous study suggested that rapid person-to-person transmission of SARS-CoV-2 may have occurred [19]. However, how easily the virus is transmitted between persons is currently unclear [18].

24 January 2020 is the Chinese New Year. It is a tradition for Chinese people that migrant workers return home before the Spring Festival. According to the mayor of Wuhan, more than 5 million people have left the city because of the Spring Festival and the outbreak of COVID-19 [20].

To control the epidemic of COVID-19, Wuhan Municipal People's Government issued a notice saying that citizens should not leave Wuhan for no special reason and the airport and train station from Wuhan corridor were temporarily closed from 23 January 2020 which was called ‘Wuhan lockdown’. In addition, Wuhan's urban bus, subway, ferry and long-distance passenger transportation were suspended [5]. Subsequently, Hubei province launched first-level response to major public health emergencies, which means movement of people among provinces, municipalities and autonomous regions was restricted [6]. Our study found that Wuhan migration scale index has dropped significantly since ‘Wuhan lockdown’ and Hubei province migration scale index has dropped significantly since Hubei province launching first-level response to major public health emergencies.

Our study also found that Wuhan migration scale index from 9 January to 22 January, 10 January to 23 January and 11 January to 24 January was correlated with the number of new confirmed COVID-19 cases per day in China except for Wuhan from 20 January to 2 February. This suggests that people who left Wuhan from 9 January to 24 January may led to the outbreak in China except for Wuhan. Meanwhile, our study found that Hubei province migration scale index from 10 January to 23 January and 11 January to 24 January was correlated with the number of new confirmed COVID-19 cases per day in China except for Hubei province from 20 January to 2 February. This suggests that people who left Hubei province from 10 January to 24 January may lead to the outbreak in China except for Hubei province.

Calculated from 24 January 2020, after a long incubation period it is 7 February 2020. Our study found that new confirmed COVID-19 cases per day in China except for Wuhan showed a downward trend from 6 February 2020 and new confirmed COVID-19 cases per day in China except for Hubei province showed a downward trend from 4 February 2020. This may indicate that ‘Wuhan lockdown’ and Hubei province launching first-level response to major public health emergencies have had a good effect on the control COVID-19 epidemic. This may also indicate the occurrence of second-generation cases because there were still new confirmed COVID-19 cases in China except for Wuhan and Hubei provinces after 7 February 2020.

In spite of the above findings, the limitations in our study should be considered. First, the migration scale of Wuhan and Hubei provinces should include migration to other countries. However, our study only included the number of new COVID-19 cases in China (including Hong Kong, Macau and Taiwan) and didn't include the number of new COVID-19 cases abroad. Second, the number of onset per day should be used in the natural process of disease research. However, the number of new confirmed COVID-19 cases per day was used in this study. Third, we did not explore the correlation between the number of moving into each province from Wuhan or Hubei and the number of new confirmed COVID-19 cases in each province, we will conduct further work to explore the correlation between the number of moving into each province from Wuhan or Hubei and the number of new confirmed COVID-19 cases in each province.

## Conclusions

Our study found that people who left Wuhan from 9 January to 22 January, and those who left Hubei province from 10 January to 24 January, led to the outbreak in the rest of China. The ‘Wuhan lockdown’ and the launching of the first-level response to this major public health emergency may have had a good effect on controlling the COVID-19 epidemic. Although new COVID-19 cases continued to be confirmed in China outside Wuhan and Hubei provinces, in our opinion, these are second-generation cases.

## Supplementary material

For supplementary material accompanying this paper visit https://doi.org/10.1017/S0950268820001119. click here to view supplementary material

## Data

The datasets used and analysed during the current study is available from the corresponding author Huijie Chen (E-mail: chj1317@126.com) on reasonable request.
